# Major Clues and Pitfalls in the Differential Diagnosis of Parathyroid and Thyroid Lesions Using Fine Needle Aspiration Cytology

**DOI:** 10.3390/medicina56110558

**Published:** 2020-10-24

**Authors:** Hwa Jeong Ha, Eun Ju Kim, Jung-Soon Kim, Myung-Soon Shin, Insup Noh, Sunhoo Park, Jae Soo Koh, Seung-Sook Lee

**Affiliations:** 1 Department of Pathology, Korea Cancer Center Hospital, Korea Institute of Radiological & Medical Sciences, Seoul 01812, Korea; cytoha@kirams.re.kr (H.J.H.); ahnjaeys@gmail.com (J.-S.K.); Shinkcch@daum.net (M.-S.S.); sunhoo@kirams.re.kr (S.P.); jskoh@kirams.re.kr (J.S.K.); 2 Convergence Institute of Biomedical Engineering and Biomaterials, Seoul National University of Science and Technology, Seoul 01811, Korea; insup@seoultech.ac.kr; 3 Division of Radiation Biomedical Research, Korea Institute of Radiological & Medical Sciences, Seoul 0182, Korea; ejkim@kirams.re.kr; 4 Radiological & Medico-Oncological Sciences, University of Science & Technology, Daejeon 34113, Korea; 5 Department of Chemical and Biomolecular Engineering, Seoul National University of Science and Technology, Seoul 01811, Korea

**Keywords:** parathyroid lesion, thyroid lesion, fine needle aspiration cytology, diagnostic pitfalls

## Abstract

Background: It is difficult to distinguish parathyroid lesions (PLs) from thyroid lesions using fine needle aspiration cytology (FNAC) because of their proximity and their similar cytomorphological features. Methods: FNAC smears of 46 patients with pathologically proven PLs that were histologically diagnosed as parathyroid adenoma (PA, *n* = 35), parathyroid hyperplasia (PH, *n* = 3), atypical parathyroid adenoma (APA, *n* = 1), and parathyroid carcinoma (PC, *n* = 7) were retrospectively reviewed and analyzed. Results: Our initial cytological diagnoses indicated correct diagnoses in 31 of 46 PL patients (67%). The 15 erroneous diagnoses were 5 patients with non-specific benign disease (11%), 4 with nodular hyperplasia of the thyroid (9%), 5 with atypical cells (11%), and 1 with a metastatic papillary thyroid carcinoma (2%). Follicular pattern, papillary structures, colloid-like material, and macrophages, which often suggest thyroid lesions, were also present in some PLs. We found that branching capillaries along the papillary structures, stippled nuclear chromatin, and frequent occurrence of naked nuclei were useful for determining a parathyroid origin. Conclusions: It is important to be aware that PLs are frequently mistaken for thyroid lesions based on FNAC. The specific and unique characteristics of PLs identified here may be helpful in diagnosis.

## 1. Introduction

Parathyroid adenomas (not parathyroid carcinomas) are very common, probably a daily finding in most endocrine clinics [1,2]. Attie et al. reported the first case in 1967, and subsequent researchers have used this term in cases in which there was intraoperative discovery of one or more enlarged parathyroid glands, without information on hyperparathyroidism [3,4,5]. Hyperparathyroidism is a cause of hypercalcemia in females who are 30 to 50 years-old [6]. The most common abnormality in most cases (80–85%) is a single parathyroid adenoma (PA), but other causes are parathyroid hyperplasia (PH), multiple PAs, and rarely parathyroid carcinoma (PC) [7,8,9]. Real-time ultrasonography can easily locate surrounding tissues, including the thyroid glands. Recently, the incidence of asymptomatic parathyroid lesions (PLs) has increased because of the increasing use of sono-guided fine needle aspiration cytology (FNAC) of the thyroid [10,11]. However, hypoechoic nodules outside the thyroid gland are not easily distinguished from lymph node enlargement or thyroid nodules in patients who have PAs or multinodular goiters in the thyroid gland [12]. In particular, when the lesion is adjacent to the back of the thyroid gland, it is not easy to distinguish thyroid nodules from parathyroid nodules [13]. Furthermore, it is very difficult to distinguish thyroid lesions and PLs based on cytological findings without clinical evidence [14,15,16].

In cases where follicular patterns from thyroid lesions or cells similar to Hürthle cells are present in cytological smears, it can be difficult to distinguish thyroid lesions from PLs. Moreover, if FNAC is performed without knowing the serum level of parathyroid hormone (PTH), it is even more difficult to judge the follicular pattern as a cytomorphological feature of a PL. At present, if a FNAC of adjacent nodules behind the thyroid gland leads to ambiguity in identifying the cells as from the thyroid or parathyroid gland, then immunohistochemistry (IHC) is performed. However, IHC analysis is not always available in FNAC material. Although it is not always easy to accurately diagnose PLs by FNAC in clinically ambiguous lesions, including the thyroid gland, parathyroid gland, and lymph nodes, it is extremely important to consider the possibility of PLs as an expectation in some clinical diagnoses. Accordingly, an accurate diagnosis of PLs requires careful examination.

In this study, we compared the cytological, nuclear, cytoplasmic, and background features of FNAC samples of histologically confirmed PLs to identify FNAC features useful for the diagnosis of PLs.

## 2. Materials and Methods

Forty-six patients with confirmed histological diagnosis of PL from January 2007 to December 2018 were identified in the database of the Korea Cancer Center Hospital. FNAC was performed in all 46 patients prior to surgical resection. The records of these 46 patients, which had histological diagnoses of PA (*n* = 35), PH (*n* = 3), atypical parathyroid adenoma (APA; *n* = 1), and PC (*n* = 7), were reviewed and compared with the corresponding cytological diagnoses.

FNAC was used to obtain cell samples via a 22–25 gauge needle with a 10 mL disposable syringe attached under negative pressure [17]. The sample was put onto 4 glass slides, smeared, and immediately fixed in 95% ethanol. To reduce false-negative diagnoses, the syringe and needle were rinsed with normal saline solution to remove residual material for making a cell block from the aspirate [18]. FNAC smear slides were stained using an automated Papanicolaou stainer (Thermo Scientific, Walldorf, Germany), and cell blocks were cut into 4-μm sections and then stained with hematoxylin and eosin. IHC staining was performed with a Bond-III automatic slide stainer (Leica Biosystems Melbourne Pty., Ltd. VIC, Melbourne, Australia) using a cell block when differential diagnosis was required. Smears were available for all 46 patients, and cell blocks were available for 45 patients.

FNAC and histological diagnoses of all 46 patients were compared, and cytological details were evaluated for the different histological groups. The cytological analysis focused on architectural, nuclear, cytoplasmic, and background features. The architectural features evaluated were follicular structures, papillary-like clusters with vascular cores, loose clusters, tight clusters, and dispersed cells. The nuclear features analyzed were stippled chromatin, nucleoli, nuclear grooves, intranuclear pseudoinclusions, and anisonucleosis. The cytoplasmic features analyzed were granularity and oxyphilic change. The background features evaluated were colloid, colloid-like material, macrophages, lymphoid cells, and bare nuclei.

This study was approved by the Institutional Review Board of the Korea Cancer Center Hospital, with a waiver of informed consent (IRB FILE No. 2018-03-006).

## 3. Results

### 3.1. Analysis of Parathyroid Lesions (PLs) Using Ultrasound-Guided FNAC

We reviewed the records of 10 males and 36 females who underwent parathyroidectomy from January 2007 to December 2018. Their ages ranged from 27 to 71 years (mean: 48.6); 12 patients were under 40 years, 14 were 41 to 50 years, and 20 were older than 51 years. The mean tumor size was 2.0 cm (range: 0.2–5.6 cm); 29 tumors were less than 2.0 cm, 10 were 2.0 to 3.0 cm, 7 were larger than 3.0 cm, and the largest was 5.6 cm. Six patients had lesions in the normal parathyroid position, 29 had lesions in the thyroid, 9 had paratracheal lesions, and 2 had lesions in the neck. Most lesions (87%) were in unexpected sites. Therefore, recognizing their unusual locations was the first step in avoiding misdiagnosis. By ultrasonographic evaluation, 33 out of 46 patients were suggested as parathyroid lesion, and the remaining 13 cases represented various sonographic diagnoses including papillary thyroid carcinoma, tuberculous lymphadenopathy, and so on (Table 1). MIBI scintigraphy was performed on 39 patients, and 36 of them showed the results compatible with parathyroid adenoma (Table 1). Although imaging work-ups may provide a guideline for preoperative diagnosis, FNAC and imaging methods would play complementary roles for diagnosis of parathyroid lesions.

### 3.2. Serum PTH and Ionized Calcium Levels

At the time of the FNAC, the levels of serum PTH (normal range: 14 to 72 pg/mL) and ionized calcium (normal range: 4.48 to 4.92 mg/dL) were available for 45 patients (Table 2). The serum PTH level was normal in 4 patients (28.5 to 52.7 pg/mL) and elevated in 41 patients (83 to more than 1900 pg/mL). The four patients within the normal limits all had parathyroid adenomas. The serum ionized calcium level was elevated in 40 patients (4.93 to 6.26 mg/dL) but were not elevated in 3 patients with parathyroid adenomas and in 1 patient with parathyroid hyperplasia.

### 3.3. Comparison of Histological and Initial Cytological Diagnoses

We reviewed the surgical resection results to analyze the relationship of cytological and histological diagnoses (Table 3). The results indicated 3 patients had PH, 35 had PAs, 1 had an APA, and 7 had PCs. Thus, PLs were correctly diagnosed by FNAC in 31 patients (67%) and misdiagnosed in 15 patients (33%). The erroneous cytological diagnoses were in five patients with benign (non-specific) growths (11%), four (9%) with nodular hyperplasia of the thyroid, five (11%) with atypical cells, and one (2%) with a metastatic papillary thyroid carcinoma (PTC). These 15 erroneous cytological diagnoses were attributed to low cellularity in 6 patients and to misinterpretation of the findings as a thyroid lesion in the other 9 patients.

### 3.4. Cytomorphological Features

The cellularity of most aspirates was moderate (28/46, 61%) or high (10/46, 22%) but was low in eight aspirates (17%). For each histologically proven PL, we reviewed cytological features, focusing on the architectural, nuclear, cytoplasmic, and background features (Table 4).

#### 3.4.1. Architectural Features

**Figure 1:**
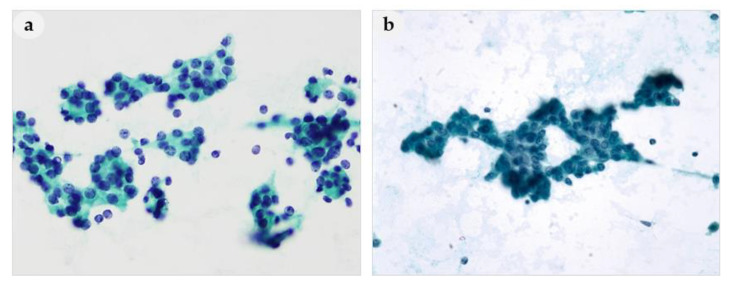
Aspirates of parathyroid adenoma. (**a**) Follicular-like pattern and (**b**) trabecular pattern mimicking follicular adenoma of thyroid. (**a**,**b**): 400× magnification.

**Figure 2:**
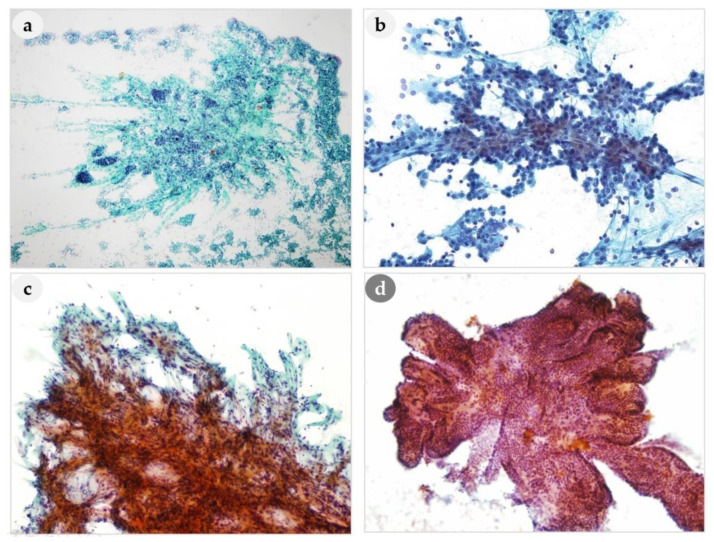
Comparison of papillary-like clusters of PLs and thyroid lesion. (**a**–**c**) Branching capillaries are aligned along the papillary structures of PLs. (**d**) Papillary-like clusters of nodular hyperplasia of thyroid. (**a**): 100×, (**b**): 400×, (**c**): 250× (**d**): 200× magnification.

Architectural features observed on FNAC smears were classified into follicular structures, papillary-like clusters with vascular cores, loose clusters, tight clusters, and dispersed cell patterns, and analyzed according to histological diagnosis. Follicular structures were present in 40% (14/35) of the PAs, but were not present in any of the patients with PH, APA, or PC (Figure 1a). Thus, a PA might be mistaken for a thyroid lesion based on FNAC (Figure 1b). Prominent papillary-like clusters with vascular cores and branching capillaries along the papillary structures (Figure 2a–c) were present in 33% (1/3) of patients with PH, 66% (23/35) of patients with PAs, and in 100% of patients with APA (1/1) and PCs (7/7). Although these features were not observed in all PLs, they are considered one of the most typical cytological characteristics of PLs. The vascular cores in the papillary fragments of PLs differed from the papillary structures in the aspirates of thyroid lesions (Figure 2d). Papillary clusters seen in nodular hyperplasia of thyroid showed tightly and regularly arranged follicular cells and usually did not show vascular cores. Moreover, the edges of papillary clusters of thyroid nodular hyperplasia were well circumscribed. On the other hand, the papillary-like structures found in parathyroid lesions contained prominent vascular cores with frequently branching vascular pattern, and frayed edges with or without clinging cells (Figure 2). Compared to papillary fragments of PLs, papillary thyroid carcinomas (PTC) tended to show rather monolayered papillary sheets, rarely with prominent vascular cores. Therefore, the presence of prominent vascular cores raises the suspicion of parathyroid lesions rather than thyroid lesions.

**Figure 3:**
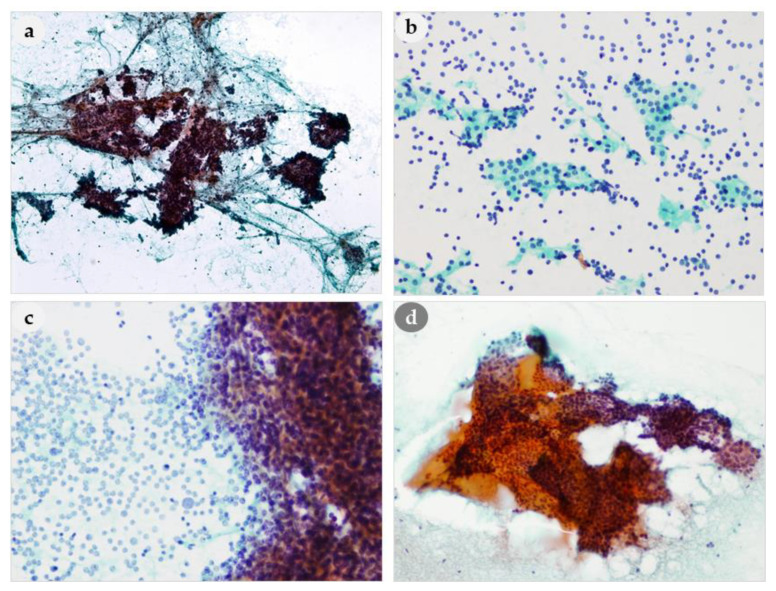
Comparison of architectural features of PLs and thyroid lesion. (**a**) Tight cohesive clusters of PL (**b**) Loose clusters and bare nuclei in the background of PL (**c**) Naked nuclei admixed with cohesive cell clusters of PL. (**d**) Monolayered sheets of benign follicular cells and colloid in the thyroid lesion. (**a**): 100×, (**b**): 200×, (**c**): 400×, (**d**): 200× magnification.

Tight clusters are also important cytomorphological features for differentiating PLs from thyroid lesions (Figure 3a). The tight clusters in the aspirate of a thyroid follicular neoplasm differed from a PL in that they appeared as small three-dimensional clusters (Figure 3d). A dispersed cell pattern was also common (Figure 3b). In one patient, the smears only showed dissociated epithelial cells without cell clusters, thus mimicking lymphoid cells. Cells arranged as cohesive cell clusters with bare nuclei in the background were characteristic (Figure 3c). If these findings are present on the same smears, they may be very helpful for the diagnosis of PL.

#### 3.4.2. Nuclear Features

**Figure 4:**
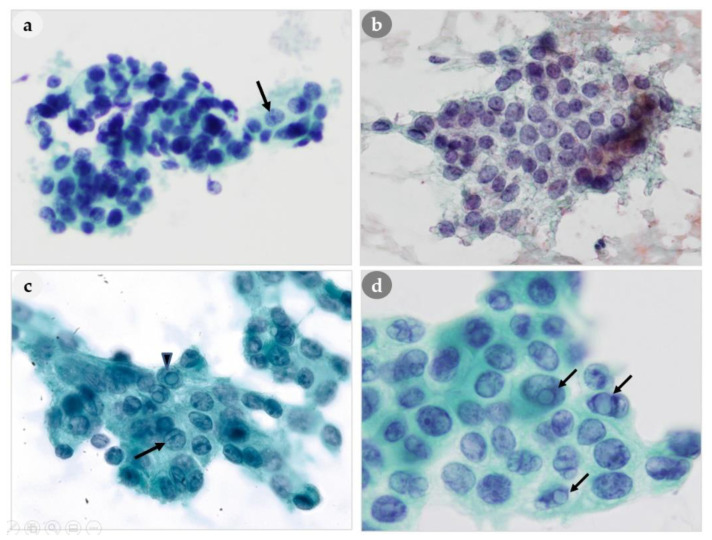
Comparison of nuclear features of PLs and thyroid lesions. (**a**) Three-dimensional clusters with stippled chromatin (arrow) of PL. (**b**) Finely granular chromatin of thyroid. (**c**) Abundant cytoplasm with intranuclear pseudoinclusions (arrowhead), nuclear grooving (arrow) of PL. (**d**) Intranuclear pseudoinclusions (arrow) of thyroid. (**a**,**b**): 400×, (**c**,**d**): 1000× magnification.

Our analysis of nuclear features indicated that a stippled chromatin pattern was present in all 46 patients, and this is regarded as an important feature for differentiating parathyroid (Figure 4a) and thyroid hyperplasia (Figure 4b). We also observed anisonucleosis, nucleoli, and nuclear grooving in some patients who had PH, PA, and PC (Table 4). We observed intranuclear pseudoinclusions (regarded as characteristics of papillary thyroid carcinoma) in 20% (7/35) of the PA patients and in 71% (5/7) of the PC patients. Nuclear grooving was also present 33% (1/3) of PH patients, 14% (5/35) of PA patients, and 43% (3/7) of PC patients. The appearance of intranuclear pseudoinclusions in PLs could lead to misidentification as a PTC, although no such misdiagnoses occurred in our study. Interestingly, our analysis indicated that the nuclear characteristics of thyroid lesions, such as intranuclear pseudoinclusions and/or nuclear grooving (Figure 4d), were also present in some cytological smears of PLs (Figure 4c); however, there were differences in the nuclear chromatin pattern, in that PLs had stippled chromatin and papillary thyroid carcinomas typically have ground-glass chromatin.

#### 3.4.3. Cytoplasmic Features

**Figure 5:**
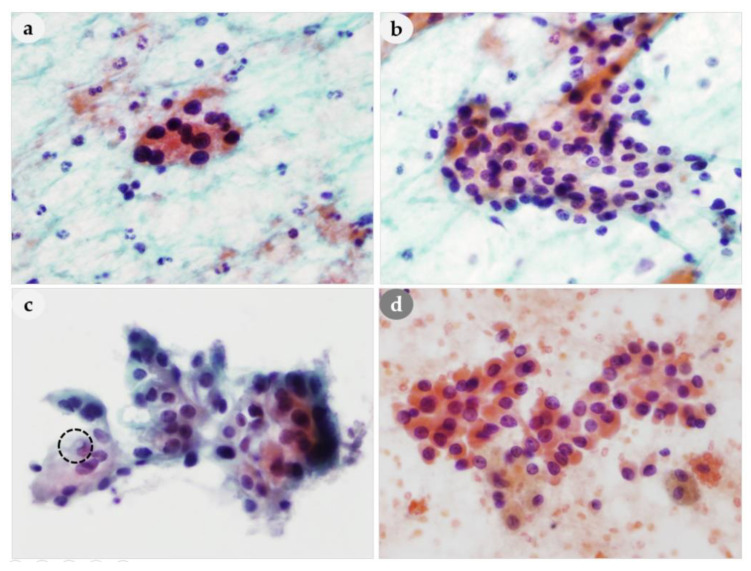
Comparison of cytoplasmic granularity of PLs and thyroid lesion. (**a**–**c**) Cytoplasmic granularity within the cluster of PLs. (**c**) A paranuclear intracytoplasmic vacuole (dotted circle) in the oxyphilic cytoplasm of PL. (**a**–**d**): 400× magnification.

We found that the cytoplasm had a characteristic granularity in all 46 patients (Figure 5a–c). However, oxyphil cells (which have abundant eosinophilic cytoplasm) were present in seven patients (Figure 5a–c), and these cells can make it difficult to differentiate a PL from a thyroid lesion with oxyphilic changes (Figure 5d). We identified paranuclear intracytoplasmic vacuoles in the oxyphilic cytoplasm in four patients (Figure 5c).

#### 3.4.4. Background and Extracellular Material

**Figure 6:**
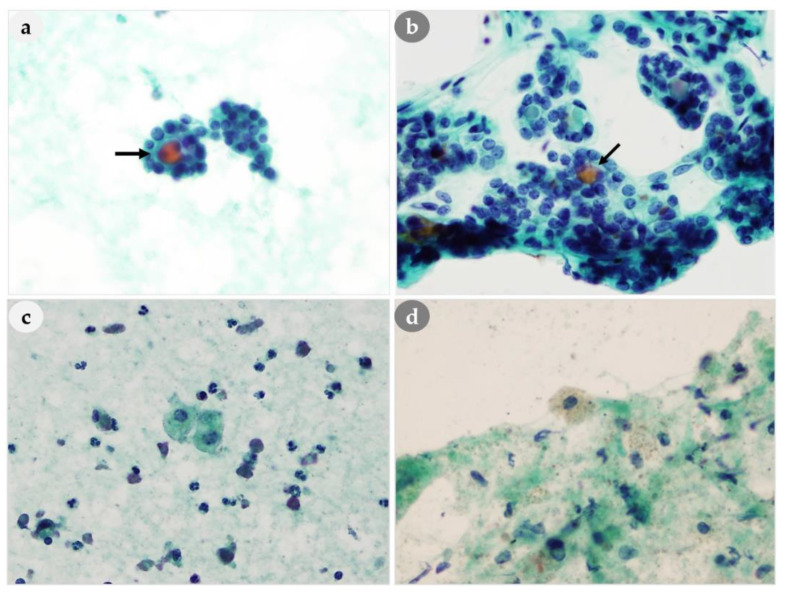
Comparison of background and extracellular material of PLs and thyroid lesions. (**a**) Inspissated colloid-like material (arrow) in the PL cell nest mimicking (**b**) the colloid (arrow) of thyroid lesion. (**c**,**d**) Macrophages and bare nuclei are found in the background. (**a**–**d**): 400× magnification.

Eleven lesions (24%) had inspissated extracellular colloid-like material in the cell nests (Figure 6a), and eight lesions (17%) had macrophages in the background (Figure 6c). They were similar to the findings in the NH of thyroid (Figure 6b,d). However, microfollicular formation containing colloid-like material can be shown in both parathyroid and thyroid lesions, but macrofollicular formation was only seen in thyroid but not in parathyroid aspirates (Figure 6b).

### 3.5. IHC Staining Results

**Figure 7:**
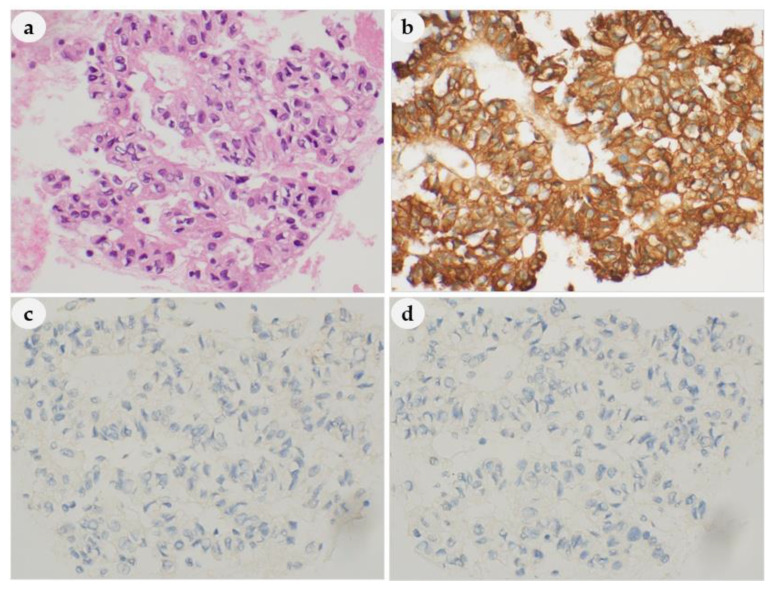
Hematoxylin and eosin (H&E) and IHC staining in the cell blocks. (**a**) H&E; (**b**) parathyroid hormone (PTH), positive; (**c**) thyroglobulin (TG), negative (**d**); thyroid transcription factor-1 (TTF-1), negative. (**a**–**d**): 400× magnification.

We performed IHC staining on cell blocks from 30 patients to determine if the origin was the thyroid or parathyroid. All 30 samples were positive for parathyroid hormone (PTH) (Figure 7b) and negative for thyroglobulin (TG) (Figure 7c) and thyroid transcription factor-1 (TTF-1) (Figure 7d), thus confirming they were PLs. Among the other samples, IHC could not be performed in six of them because of the low cellularity; PLs were diagnosed in five patients without performing IHC; and IHC was not performed at the time of FNAC in the other five patients, because PL was not suspected.

## 4. Discussion

There are difficulties in the diagnosis of PL from FNAC, because the thyroid and parathyroid are adjacent and have similar cellular morphology [19,20,21,22]. In particular, the differential diagnosis of a parathyroid tumor by FNAC is difficult if PL is not suspected based on other clinical and radiological findings. This confusion is due to the presence of papillary-like architectures, microfollicular cells, macrophages, inspissated colloid-like material, oxyphilic cytoplasm, and naked nuclei in FNAC. We therefore examined the cytological characteristics of PLs in an effort to identify the possible causes of errors in the differential diagnosis of PLs from cytologically similar thyroid lesions.

Two reviews of this subject reported poor diagnostic sensitivities for FNAC (65% and 40.4%), whereas Halbauer et al. reported that FNAC had a diagnostic sensitivity of 86% [19,23,24]. Our results showed a 67% agreement in the diagnosis of PL from cytology and histology. We also found that erroneous diagnoses were most common for benign (non-specific) lesions (11%; 5/46) and atypical cells (11%; 5/46), followed by nodular hyperplasia of thyroid (9%; 4/46), and metastatic papillary thyroid carcinoma (2%; 1/46). The primary clinical concern is the capacity to differentiate PLs from thyroid lesions.

Our results indicated that the presence of follicular structures, oxyphilic cells, macrophages, and colloid-like material in smears was most likely to lead to misinterpretation of a PL as a thyroid lesion. In their reviews, Kini et al. and Abati et al. concluded that macrophages and inspissated colloid-like material were specific to thyroid lesions [23,24,25]. However, we found these in 12% and 18% of our PL cases, respectively; Bondeson et al. reported them in 21% and 10% of lesions, respectively; and Tseng et al. reported them in 24% and 9% of lesions, respectively [11,23,25]. Changes in Hürthle cells in thyroidal follicular cells appear similar to changes in oxyphil cells in PLs, and this could lead to misinterpretation [8,25,26]. Some studies of the parathyroid showed that colloid-like material and the presence of macrophages, particularly macrophages with cytoplasmic hemosiderin, cannot be used to differentiate thyroid and parathyroid tumors [23,24,25]. The existence of colloid-like substances and/or macrophages is obviously insufficient to exclude a PL [6]. Our study also indicated that many patients had follicular architecture (30%), colloid-like material (24%), macrophages (17%), and oxyphilic cytoplasm (15%). In addition, FNAC led to misdiagnosis in three patients without clinical suspicion of PLs as nodular hyperplasia of the thyroid. We found it was especially difficult to differentiate a PL from a thyroid lesion when microfollicular features were present. Thus, use of these findings could be a major pitfall that prevents the correct diagnosis of PL. In terms of architectural features, many of our patients had papillary-like clusters with vascular cores (70%), loose clusters (85%), tight clusters (65%), and dispersed cells (50%). The papillary structures of PLs differ from those of papillary thyroid carcinoma in that the PLs have vascular cores. Thus, the presence of prominent papillary-like clusters with vascular proliferation attached within epithelial cells, loose or tight clusters, scattered cells, and the absence of definitive thyroid tumor cells were all useful clues to a parathyroid origin.

We found variable amounts of stippled chromatin in the nuclei of all 46 patients. Furthermore, the nuclei of PLs were more hyperchromatic than those of thyroid lesions, such as papillary thyroid, nodular hyperplasia, and follicular neoplasm. In addition, although intranuclear pseudoinclusions are an important diagnostic clue in FNAC of the thyroid [27], we identified intranuclear pseudoinclusions in 20% (7/35) of PLs with a histological diagnosis of PA and in 71% (5/7) of PLs with a histological diagnosis of PC. There were some previous reports that intranuclear inclusions were not present in cytological preparations of PLs [11,25,28], although Goellner et al. and Bondeson et al. reported intranuclear inclusions in parathyroid neoplasms [27,29]. Our study confirmed that intranuclear inclusions may occur in PLs and thus stressed the need for clinicians to consider thyroid lesions and parathyroid diseases when intranuclear inclusions are evident in FNAC.

Another important feature is the presence of a finely granular eosinophilic cytoplasm, which was also present in all 46 cases. This feature could be misinterpreted as a thyroid Hürthle cell neoplasm. In fact, 40 of 46 PLs (87%) had naked nuclei in the background on FNAC. Importantly, when considering the possibility of a parathyroid neoplasm, the appearance of numerous naked nuclei in the background may help [30]. Dimashkieh and Krishnamurthy [19] also reported that the presence of numerous naked nuclei, rather than thyroid follicular cells, with a prominent vascular network, epithelial cells, and stippled chromatin were indicative of a parathyroid origin [21,31,32].

Our retrospective analysis suggests the possibility that a PL should not be excluded in examinations of thyroid lesions, even if not supported by clinical or radiographic findings. It is important to comprehensively assess findings that are valuable in the differential diagnosis, because many of the cytological findings of parathyroid tumors are similar to those of thyroid tumors, and there is no single unique diagnostic finding. Although the literature on the subject of parathyroid FNAC is limited, most authors stress that there is no specific diagnostic criterion that accurately differentiates PLs from thyroid lesions, and that diagnosis should consider a variety of cytomorphological characteristics [29,30]. Bondeson et al. and Absher et al. reported that the most useful criteria for identification of parathyroid cells may be the presence of naked nuclei, nuclear overlapping, nuclear molding, three-dimensional fragments, cohesive cell clusters, and mast cells [29,33].

Our analysis indicated the appearance of papillary-like clusters with prominent vascular cores, three-dimensional clusters, nuclei with stippled chromatin, cytoplasmic granularity within the cluster, naked nuclei in the background, and tight clusters were helpful in the diagnosis of PLs from FNAC.

Finally, IHC analysis can help differentiate parathyroid and thyroid cells [34]. Although neuroendocrine markers such as chromogranin-A, synaptophysin, and CD56 are commonly used, they have limitations in specificity [35]. Recently GATA-3 and parafibromin have been known to have a role in the pathogenesis of parathyroid diseases, and these markers have been attempted to be used in the diagnostic field of parathyroid tumors. However, the role of these markers needs to be further elucidated [35]. A panel of PTH and TG or TTF-1 is considered to be the only reliable tool in separating parathyroid from thyroid cells [35]. Furthermore, the utility of using needle rinse PTH measurements to confirm suspected PLs is a practical way to help distinguish PLs from thyroid proliferation [36]. Nevertheless, in the literature and reviewers’ experience, the reliable distinction is impossible without the use of IHC, either on CBs or direct smears, with parathormone and TG/TTF-1 as crucial markers. The modified scrape cell block (SCB) technique also demonstrated strong immunosensitivity preservation and contributed to a correct cytological diagnosis with conventional smears in 97.6 percent of all FNA [37]. Nonetheless, IHC is not always possible when the cell blocks have low cellularity or if the cells have low storage levels of PTH [36,38,39]. Therefore, a cytomorphological approach is still valuable.

## 5. Conclusions

Our study identified important diagnostic clues and pitfalls that should be considered in the differential diagnosis of PLs and thyroid lesions based on FNAC. The presence of follicular structures, colloid-like material, or macrophages in FNAC samples are major pitfalls that can lead to the misdiagnosis of a PL as a thyroid lesion. However, a stippled chromatin pattern of nuclei, papillary-like clusters with vascular cores and clinging epithelial cells, high prevalence of naked nuclei, and tight clusters and/or loose clusters are useful diagnostic clues that favor a parathyroid origin. The similar locations and cytological findings of PLs and thyroid lesions in FNAC complicate the differential diagnosis. Thus, clinicians should consider the major pitfalls and diagnostic clues identified here.

## Figures and Tables

**Table 1:** Clinical findings of parathyroid lesions in 46 cases.

	Characteristics	No. of Cases (%)
Gender	Male	10 (22)
	Female	36 (78)
Age (mean age 48.6)	≤40	12 (26)
	41–50	14 (30)
	≥51	20 (44)
Size of the lesion (range 0.2–5.6 cm)	<2.0 cm	29 (63)
	2.0–3.0 cm	10 (22)
	>3.0 cm	7 (15)
Location	Normal parathyroid position	6 (13)
	Intrathyroidal	3 (6)
	Posterior to thyroid gland	16 (35)
	Inferior to thyroid gland	10 (22)
	Paratracheal	9 (20)
	Neck	2 (4)
Ultrasonography	Parathyroid lesion	33 (72)
	Nodular hyperplasia of thyroid	6 (13)
	Papillary thyroid carcinoma	2 (4)
	Lymph node metastasis	2 (4)
	TB lymphadenopathy	1 (2)
	Suture granuloma	1 (2)
	Indeterminate nodule of thyroid	1 (2)
MIBI scintigraphy	Parathyroid adenoma (PA)	36 (92)
	No evidence of PH or PA	2 (5)
	Adenomatous goiter, more likely than PA	1 (3)
	Not done	7

No., number; TB, tuberculosis; PH, parathyroid hyperplasia.

**Table 2:** Serum PTH and calcium levels according to histological diagnoses.

Histologic Diagnosis No. of Cases (%)	PTH Level No. of Cases (%)		Ionized Calcium Level No. of Cases (%)	
	High	Normal	High	Normal
PH, *n* = 3 (%)	3 (100)	0 (0)	2 (67)	1 (33)
PA, *n* = 34 (%)	30 (88)	4 (11)	31 (91)	3 (9)
APA, *n* = 1 (%)	1 (100)	0 (0)	1 (100)	0 (0)
PC, *n* = 7 (%)	7 (100)	0 (0)	6 (86)	1 (14)
Total, *n* = 45 (%)	41 (91)	4 (9)	40 (89)	5 (11)

PTH, parathyroid hormone; PH, parathyroid hyperplasia; PA, parathyroid adenoma; APA, atypical parathyroid adenoma; PC, parathyroid carcinoma; No., number.

**Table 3:** Comparison of initial cytologic and histologic diagnoses.

Initial FNAC Diagnosis No. of Cases (%)	Histological Diagnosis No. of Cases (%)			
	PH *n* = 3 (%)	PA *n* = 35 (%)	APA *n* = 1 (%)	PC *n* = 7 (%)
Parathyroid neoplasm				
31 (67)	2 (67)	21 (60)	1 (100)	7 (100)
Benign (non-specific)				
5 (11)	0 (0)	5 (14)	0 (0)	0 (0)
Nodular hyperplasia of thyroid				
4 (9)	1 (33)	3 (9)	0 (0)	0 (0)
Atypical cells				
5 (11)	0 (0)	5 (14)	0 (0)	0 (0)
Metastatic PTC				
1 (2)	0 (0)	1 (3)	0 (0)	0 (0)

PH, parathyroid hyperplasia; PA, parathyroid adenoma; APA, atypical parathyroid adenoma; PC, parathyroid carcinoma; Metastatic PTC, metastatic papillary thyroid carcinoma; No., number.

**Table 4:** Cytologic features in aspirates according to the histologic diagnosis.

Cytologic Characteristics		Histologic Diagnosis No. of Cases (%)				
		PH *n* = 3 (%)	PA *n* = 35 (%)	APA *n* = 1 (%)	PC *n* = 7 (%)	Total *n* = 46 (%)
Architecture	Follicular structures	0 (0)	14 (40)	0 (0)	0 (0)	14 (30)
	Papillary-like cluster with vascular core	1 (33)	23 (66)	1 (100)	7 (100)	32 (70)
	Tight clusters	2 (67)	21 (60)	1 (100)	6 (86)	30 (65)
	Loose clusters	3 (100)	28 (80)	1 (100)	7 (100)	39 (85)
	Dispersed cells	1 (33)	18 (51)	0 (0)	4 (57)	23 (50)
Nuclei	Anisonucleosis	1 (33)	17 (49)	0 (0)	6 (86)	24 (52)
	Stippled chromatin	3 (100)	35 (100)	1 (100)	7 (100)	46 (100)
	Nucleoli	2 (67)	22 (63)	0 (0)	6 (86)	30 (65)
	Intranuclear pseudoinclusions	0 (0)	7 (20)	0 (0)	5 (71)	12 (26)
	Nuclear grooving	1 (33)	5 (14)	0 (0)	3 (43)	9 (20)
Cytoplasm	Vacuolation	0 (0)	2 (6)	0 (0)	2 (29)	4 (9)
	Granularity	3 (100)	35 (100)	1 (100)	7 (100)	46 (100)
	Oxyphilic	0 (0)	7 (20)	0 (0)	0 (0)	7 (15)
Background	Colloid-like material	1 (33)	7 (22)	0 (0)	3 (43)	11 (24)
	Macrophages	1 (33)	4 (13)	0 (0)	3 (43)	8 (17)
	Bare nuclei	3 (100)	29 (83)	1 (100)	7 (100)	40 (87)

PH, parathyroid hyperplasia; PA, parathyroid adenoma; APA, atypical parathyroid adenoma; PC, parathyroid carcinoma; No., number.
